# Comment on Drozdowska-Szymczak et al. Incidence and Risk Factors of Cholestasis in Newborns with Hemolytic Disease—A Case-Control Study. *J. Clin. Med.* 2024, *13*, 3190

**DOI:** 10.3390/jcm13237047

**Published:** 2024-11-22

**Authors:** Jonas Teng, Björn Fischler, Kajsa Bohlin, Marie Reilly, Eleonor Tiblad

**Affiliations:** 1 Division of Pediatrics, Department of Clinical Science, Intervention and Technology (CLINTEC), Karolinska Institutet, 141 52 Huddinge, Sweden; bjorn.fischler@ki.se (B.F.); kajsa.bohlin@ki.se (K.B.); 2 Department of Pediatrics, Södertälje Hospital, 152 86 Södertälje, Sweden; 3 Department of Pediatrics, Karolinska University Hospital, 141 86 Stockholm, Sweden; 4 Department of Neonatology, Astrid Lindgren Children’s Hospital, Karolinska University Hospital, 141 86 Stockholm, Sweden; 5 Department of Medical Epidemiology & Biostatistics, Karolinska Institutet, 171 77 Stockholm, Sweden; marie.reilly@ki.se; 6 Clinical Epidemiology Unit, Department of Medicine Solna, Karolinska Institutet, 171 77 Stockholm, Sweden; eleonor.tiblad@ki.se

We read with interest the recently published article in the *Journal of Clinical Medicine*, by Drozdowska-Szymczak et al., entitled “Incidence and risk factors of cholestasis in newborns with hemolytic disease—a case-control study” [1], because the topic is of clinical importance and further research is warranted. However, we have several objections regarding the methodology and interpretation of the results that we would like to share with you.

In this retrospective observational study, the researchers investigate the risk factors and incidence rate of cholestasis among newborn infants with hemolytic disease of the fetus and newborn (HDFN) during a four-year period in a single center. They compare cholestatic and non-cholestatic infants with HDFN, defining cholestasis as conjugated bilirubin exceeding 1 mg/dL. The authors report that the risk factors for cholestasis are prematurity, Rh or Kidd immunization, and the need for intrauterine erythrocyte transfusion.

We have the following concerns regarding this article:

First, the definition used for cholestasis in this population may not be optimal. The definition used is based on the level recommended to be regarded as abnormal by international guidelines from the North American and European Societies of Pediatric Gastroenterology, Hepatology and Nutrition (NASPGHAN and ESPGHAN) [2]. This definition is recommended for the evaluation of infants with prolonged jaundice for the early detection of, for example, biliary atresia. Using this definition in infants with HDFN is problematic. HDFN is a severe hemolytic condition with often extremely high unconjugated bilirubin levels, and this often causes a concomitant slight increase in conjugated bilirubin that is not due to cholestasis. Using a definition without a criterion of conjugated bilirubin to also exceed 20% of the total level will probably cause misclassification: infants that clinically would not be regarded as cholestatic will be classified as cholestatic using this definition.

Second, the authors do not report the cause or modes of delivery. This is almost always reported in neonatal research.

More severe HDFN more often leads to an iatrogenic delivery, such as a caesarian section or induced vaginal delivery. The slightly more (but significant) preterm births among the cholestatic cases is most likely due to the severity of HDFN in this group. Hence, the cholestasis is most likely due to more severe HDFN in these infants and the prematurity confounding by indication. Why did the authors not report the cause and mode of delivery and the possible bias of iatrogenic preterm delivery?

Third, the authors have included ABO immunizations in their study group. ABO immunization does not cause severe HDFN during pregnancy, since the antigens develop late in fetal life and thus do not result in the need for intrauterine transfusion. Previous studies regarding cholestasis in infants with HDFN do not include ABO immunizations since it is very unlikely that it will cause this complication [3,4]. If the ABO infants are removed from Table 1 in the article by Drozdowska-Szymczak et al. [1], and Fisher’s exact tests are recalculated, all of the significant findings disappear except for Kidd immunization. The significant finding for Kidd is based on just five cases in the cholestasis group and one in the non-cholestasis group, providing very weak clinical evidence. The lack of significant findings when the ABO group is excluded, as in other studies, suggests that the reported results are largely spurious, and in any case, they cannot be compared with other published work.

Fourth, the authors compared “first bilirubin tests”, which means comparing postnatal values in ABO infants (when discovered because of jaundice during postnatal care) to umbilical or very early samples from the prenatally diagnosed infants with HDFN.

To summarize, we are concerned regarding the definition of cholestasis used, the lack of information on the mode of delivery, the conclusion that prematurity is a risk factor for cholestasis, the inclusion of ABO-infants in the study population, and the comparison of “first bilirubin tests” that could have been obtained at different ages in the two groups.

We believe clarifications regarding these issues are important for the credibility of the research and the validity of any interpretations.
